# One center experience with a personalized frozen-thawed embryo transfer in patients with recurrent implantation failure

**DOI:** 10.1007/s10815-023-02835-7

**Published:** 2023-06-01

**Authors:** Philippos Edimiris, Cornelius Doehmen, Dunja Maria Baston-Buest, Jan-Steffen Kruessel, Alexandra Petra Bielfeld

**Affiliations:** 1 Department of OB/GYN and REI (UniKiD), Medical Center University of Duesseldorf, Moorenstraße 5, 40225 Duesseldorf, Germany; 2 Kinderwunschzentrum Niederrhein, Madrider Str. 6, 41069 Moenchengladbach, Germany

**Keywords:** Recurrent implantation failure, Endometrial receptivity analysis, Window of implantation, Programmed frozen-thawed embryo transfer

## Abstract

**Purpose:**

Displaced endometrial receptivity has been discussed as a possible cause of recurrent implantation failure in patients undergoing assisted reproductive technology. The aim of this study was to document our experience with the endometrial receptivity analysis in patients with recurrent implantation failure.

**Methods:**

This retrospective cohort study, conducted at the Fertility Centre of the University Hospital, Duesseldorf Germany, presents the results of the endometrial receptivity analysis in 67 patients with recurrent implantation failure and compares the clinical outcome between these 67 patients who underwent a personalized frozen-thawed embryo transfer guided by the results of the endometrial receptivity analysis and 32 patients with recurrent implantation failure who performed a standardized frozen-thawed embryo transfer.

**Results:**

The data analysis revealed a displaced endometrial receptivity in 73% (49/67) of all tested patients. Out of these patients, 24% (12/49) were early receptive, 74% (36/49) were pre-receptive, and 2% (1/49) were post-receptive. Comparison of pregnancy rate, clinical pregnancy rate, and live-birth rate between personalized (49%, 39%, 27%, respectively) and standardized embryo transfer (44%, 31%, 19%, respectively) reveals no statistically significant difference. In both groups, patients had an average of four unsuccessful embryo transfers.

**Conclusion:**

In this cohort of patients with recurrent implantation failure, the endometrial receptivity analysis showed a high incidence of displaced endometrial receptivity. However, a personalized embryo transfer did not increase reproductive outcome. Displaced endometrial receptivity might not be the main cause for recurrent implantation failure in this cohort.

**Supplementary information:**

The online version contains supplementary material available at 10.1007/s10815-023-02835-7.

## Introduction

Despite tremendous achievements in assisted reproductive techniques (ART) in the last decades, approximately 5–10% of patients continue to suffer from recurrent implantation failure (RIF) [1, 2].

So far, a general consensus for the definition of RIF is still missing. Some authors only count the number of unsuccessful cycles or the number of embryos transferred, whereas others combine the number of unsuccessful cycles and embryos transferred with additional factors like maternal age and/or embryo quality [2, 3].

One of the main reasons for RIF are embryonic chromosomal abnormalities [4]. Further etiological causes for RIF are maternal factors such as uterine polyps, fibroids, anatomical pathologies of the uterus [5], infections leading to chronic endometritis [6], immunological factors such as cytokines, and male factors [2].

A successful implantation depends on a functional and therewith receptive endometrium and a good quality embryo [7].

Progesterone is essential in this process for the establishment of the endometrial receptivity [8]. However, endometrial receptivity exists only for a short period of time and a successful implantation depends on a synchrony between the development of a receptive endometrium and the embryo [9].

In the context of ART, the synchrony may be impaired. Evidence of an asynchrony between the endometrium and the embryo is provided, on the one hand, by a premature rise in serum progesterone that decreases the pregnancy rate in a fresh embryo transfer cycle [10]. On the other hand, in a programmed frozen-thawed embryo transfer (FET), pregnancy rates are significantly influenced by the duration of progesterone administration [11]. Thus, another reason for RIF might be an individual displaced endometrial receptivity due to an interindividual varying requirement of progesterone exposure time to accomplish adequate receptivity.

Microarray and sequencing technology have enabled the identification of the human endometrial transcript profile [12]. The transcriptomic pattern shows significant changes during the different phases of a menstrual cycle and allows the identification of a transcriptomic signature indicating receptivity of the endometrium [13]. In 2011, the Endometrium Receptivity Analysis (ERA^®^) was introduced. It evaluates the expression of 248 genes in endometrial tissue representing the endometrial receptivity profile [14]. Nowadays, the analysis is performed by Next Generation Sequencing (NGS). The endometrial receptivity analysis determines the personalized duration of progesterone exposure needed to reach a receptive status in the endometrium. The reproducibility of the test results is 100% consistent up to 36 months [15].

In a therapy-naïve patient population, a personalized embryo transfer guided by the result of an endometrial receptivity analysis does not increase reproductive outcome [16, 17]. However, in patients with RIF, the benefit of endometrial receptivity analysis still remains controversial [18]. All studies, that have investigated the benefit of an endometrial receptivity analysis in a RIF population are retrospective and have only a small number of cases [19–25]. In addition to the ERA^®^, several other endometrial receptivity tests on the basis of gene expression are now commercially available, like ERPeak^®^ (CooperSurgical^®^, Malov, Denmark), ERT (Diágnistica Longwood, Zaragoza, Spain), ER Map^®^ (iGLS, Genetics, Alicante, Spain) and beReady^®^ (Competence Centre on Health Technologies, Tartu, Estonia) [26]. Convincing data regarding the benefit of those tests are also still lacking.

Thus, the question remains whether a displaced endometrial receptivity can be causative for RIF, and whether a personalized embryo transfer guided by an endometrial receptivity analysis increases the reproductive outcome in a cohort of RIF patients.

In our study we therefore analyzed results of the endometrial receptivity analysis in RIF patients. Moreover, we investigated the reproductive outcome of patients with RIF after either a personalized embryo transfer guided by the endometrial receptivity analysis or a standardized embryo transfer in a programmed FET.

## Material and methods

### Study design and population

The present study is a retrospective analysis at the interdisciplinary Fertility Centre of the University Hospital Duesseldorf, Germany (UniKiD). The observation period was between March 2016 and June 2021. The study was approved by the University Ethics committee. The trial registration number is 2019–797.

In total, the endometrial receptivity analysis was performed in 134 patients with implantation failure during the study period. The indication to perform the test was left to the treating physician.

**Fig. 1:**
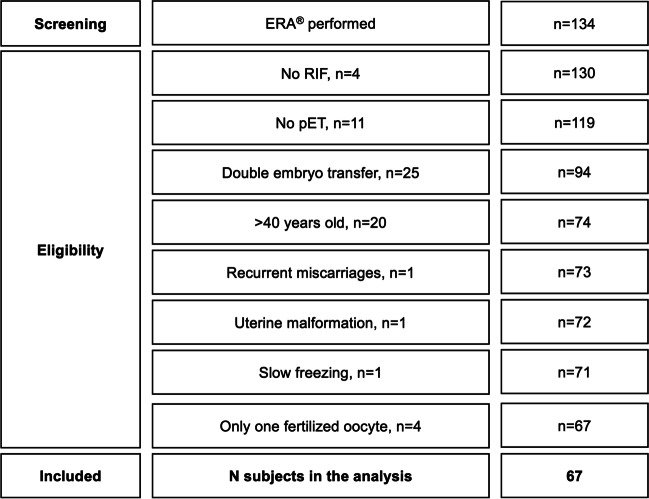
Study flow chart of the group of patients who performed an endometrial receptivity analysis. RIF: recurrent implantation failure, ERA^®^: endometrial receptivity analysis, pET: personalized embryo transfer

Inclusion criterium for the statistical analysis was RIF. RIF was diagnosed when no clinical pregnancy occurred after at least two embryo transfers with a total of three good quality embryos. The definition of RIF is derived from the center’s own data, which show that for women younger than 40 years of age, there is a cumulative clinical pregnancy rate of 93.9% after the transfer of three ideal embryos. All patients who did not meet the criteria for RIF were excluded from further analysis (*n* = 4). Further exclusion criteria were: no consecutive personalized embryo transfer (*n* = 11), double embryo transfer (*n* = 25), age older than 40 years at the time of oocyte retrieval (*n* = 20), only one oocyte fertilized (*n* = 4), recurrent miscarriages with ≥ 3 abortions (*n* = 1), uterine malformations (uterus bicornis unicollis with non-communicating atretic left uterine horn in the state after metroplasty, according to Strassmann, *n* = 1), and slow freezing (*n* = 1). Finally, 67 RIF patients were considered for the analysis (case group). See Fig. 1.

For the control group, which consisted of 32 subjects, only patients with RIF who had not undergone endometrial receptivity analysis were selected. Inclusion and exclusion criteria of the control group correspond to those of the case group.

Reproductive outcomes of the case group after personalized embryo transfer were compared to reproductive outcomes in the control group after standardized embryo transfer.

Prior to endometrial receptivity mock cycle, a vaginal ultrasound was performed for all patients. There was no evidence for adhesions, hydrosalpinx, submucous polyps, or fibroids in any patient. Our routine hormonal check-up showed normal values for each parameter examined (thyroid-stimulating hormone, prolactin, follicle stimulating hormone (FSH), estradiol).

### Protocol and sampling

The mock cycle for performing the endometrial receptivity analysis was performed following a hormonal replacement protocol: estradiol patches (Estramon^®^; Hexal AG, Holzkirchen, Germany) were started from the 1st day of the menstrual cycle in an increasing dosage from 100 μg on days 1 to 7, 200 μg on days 8 to 11, and 400 μg from the 12^th^ day. Patches were changed every 48 h.

On cycle days 12 to 14, a vaginal ultrasound examination was performed, combined with estradiol and progesterone serum analysis.

When the endometrial lining was ≥ 6 mm and the progesterone level was < 0.5 ng/ml, progesterone supplementation was started. For the progesterone supplementation, progesterone vaginal gel (Crinone^®^ 8% 90 mg, Merck KGAA, Darmstadt, Germany), 50 mg daily of oral dydrogesterone (Duphaston^®^, Mylan Germany GmbH, Troisdorf, Germany), 600 mg of vaginal micronized progesterone (utrogest^®^, Besins Healthcare, Berlin, Germany or Famenita^®^, Exeltis Germany GmbH, Ismaning, Germany), or subcutaneous progesterone (Prolutex^®^, Marckyrl Pharma, Papenburg, Germany) were used.

Following our FET cycle standard regimen, the endometrial biopsy was performed approximately 108–120 h after starting progesterone supplementation. Endometrial biopsy was performed with a Pipelle catheter (Gynetics^®^, Lommel, Belgium) and the sample proceeded as indicated by the manufacturer.

The test result differs in receptive and non-receptive. Non-receptive is divided into early receptive (endometrial receptivity 12 h later), pre-receptive (endometrial receptivity > 12 h delayed), and post-receptive results. If the test result was pre-receptive and the receptivity could not be assumed to be in the following 24 h after the initial biopsy, a second biopsy was recommended by the manufacturer. The same scheme was applied with an endometrial sampling 24 h later to predict a precise displacement and therewith a needed duration time of progesterone exposure to reach the individual endometrial receptivity.

### Quality of embryos

The quality of the embryos was assessed by using morphological characteristics. A cleavage stage embryo with good quality includes four or five blastomeres on day 2, and at least seven blastomeres on day 3, with less than 20% fragmentation and no signs of multinucleation [27]. For the classification of blastocysts, we used the alphanumeric system published by Gardner and Schoolcraft [28]. A high-quality blastocyst was defined as a blastocyst, whose grade of expansion is ≥ 3 and inner cell mass and trophectoderm is characterized as grade A and B. PGT-A could not be performed due to the German embryo protection law.

### Embryo transfer

Only cycles in which a single embryo transfer was performed were selected for the study. For the analysis, only the subsequent FET cycle after receiving the test result was included in the study. Endometrium preparation in the group of patients with a personalized embryo transfer was performed according to the patients’ mock cycle. Embryo transfer of cleavage stage embryos (day 2 or day 3) and blastocysts was performed after the recommended time of progesterone exposure. Endometrial preparation was performed in the control group congruently to the group with receptivity testing.

### Definitions of outcomes

The definitions of the terms used to describe clinical outcomes were based on the International Glossary on Infertility and Fertility Care [29]. Our primary outcome was clinical pregnancy rate (CPR), secondary outcomes were pregnancy rate, live birth rate (LBR), rate of biochemical pregnancy loss, and clinical miscarriage rate. Pregnancy was determined as a positive serum level of beta-human chorionic gonadotropin (hCG) at 9 or 10 days after tranfer of a blastocyst, and 11 or 12 days after transfer of a cleavage stage embryo. Clinical pregnancy was the ultrasonographic visualization of one or more intrauterine gestational sacs. Biochemical pregnancy loss was defined as the short-term detection of a beta-hCG without sonographic evidence of a gestational sac. Clinical miscarriage was specified as the spontaneous loss of a clinical pregnancy. Pregnancy rate, CPR, and LBR were expressed as rate per embryo transfer. Clinical miscarriage rate was expressed as rate per clinical pregnancy. Biochemical pregnancy loss rate was expressed as rate per pregnancy. Age, body mass index (BMI), anti-müllerian hormone (AMH), number of previous failed cycles, and days between last biopsy and embryo transfer were reported as mean ± standard deviation. The proportion of embryo transfer using blastocyst and embryo of good quality, as well the proportion of patients with primary sterility, are reported in percentage. We performed further clinical outcome analyses in which we subdivided the case group by receptive and non-receptive patients.

### Statistical analysis

Data were analyzed using IBM SPSS Statistics 27. For categorical variables, Pearson’s Chi-squared test was used, or Fisher’s exact test when one of the expected counts was less than 5. Continuous variables were tested for normal distribution using Shapiro–Wilk-test. In independent samples t-test was used for continuous variables with normal distribution. Mann–Whitney U test was used for continuous variables without a normal distribution. A p-value of < 0.05 was considered significant.

An a priori power analysis was performed. Assuming an increase in CPR from 30% after standardized embryo transfer to 40% after personalized embryo transfer, 356 subjects in both groups must be assessed to provide evidence of significance.

## Results

In this retrospective cohort study, we first analyzed the endometrial receptivity status in a population of RIF patients and subsequently examined the benefit of the test result by comparing the reproductive outcome of 67 RIF patients who underwent a personalized embryo transfer vs. 32 RIF patients who underwent a standardized embryo transfer.

Patients characteristics like age, BMI, and AMH, as well as characteristics concerning fertility history like number of failed embryo transfers, and proportion of patients with miscarriages and deliveries did not significantly differ between both groups (Table 1).

**Table 1:** Patient characteristics and clinical outcome after a personalized frozen-thawed embryo transfer (pET) compared to patients undergoing standardized frozen-thawed embryo transfer (standET)

	pET *n* = 67	standET *n* = 32	Odds Ratio (95% CI)^d^	*p*-value
Patient characteristics				
Age, years	35 (± 3)	36 (± 3)	—	0.73^b^
Body mass index, kg/m^2^	23 (± 5)	22 (± 5)	—	0.26^b^
AMH, ng/mL	3.1 (± 2.4)	3.1 (± 2.5)	—	0.94^b^
ICSI rate, *n* (%)	59 (88%)	29 (91%)	—	> 0.99^c^
Previous spontaneous clinical miscarriages, *n* (%)	16 (24%)	3 (9%)	—	0.087^a^
Previous ectopic pregnancies, *n* (%)	7 (10%)	0 (0%)	—	0.093^c^
Previous deliveries, *n* (%)	6 (9,0%)	2 (6%)	—	0.64^a^
Number of previous failed embryo transfers	4 (± 2)	4 (± 1)	—	0.12^b^
Time between scratching and transfer, days	116 (± 76)	—	—	—
Clinical outcome				
Pregnancy rate, *n* (%)	33 (49%)	14 (44%)	1.2 (0.5–2.9)	0.61^a^
Clinical pregnancy rate, *n* (%)	26 (39%)	10 (31%)	1.4 (0.6–3.4)	0.47^a^
Live birth rate, *n* (%)	18 (27%)	6 (19%)	1.6 (0.6–4.5)	0.38^a^
Clinical miscarriage rate, *n* (%)	8/26 (31%)	4/10 (40%)	0.7 (0.1–3.0)	0.60^a^
Biochemical pregnancy loss rate, *n* (%)	7/33 (21%)	4/14 (29%)	0.7 (0.2–2.8)	0.59^a^
Embryo transfer using blastocyst, *n* (%)	53 (79%)	24 (75%)	—	0.65^a^
Good quality embryo rate, *n* (%)	49 (73%)	27 (84%)	—	0.22^a^

Numerical variables are reported as M (± SD) and categorical variables as n (%); *p*-value controlled with:

^a^Chi-squared test, ^b^ Mann–Whitney U test, ^c^ Fisher´s exact test; ^d^ Odds Ratio for pET vs. standET are only computed for relevant clinical outcomes

The endometrial receptivity analysis revealed an endometrial receptivity in 27% (18/67) of all patients. Out of the 73% (49/67) patients with a non-receptive result, 24% (12/49) were early receptive, 74% (36/49) were pre-receptive, and 2% (1/49) were post-receptive. In 6% (4/67) of patients a second biopsy was necessary. In a total of 36 patients the endometrial biopsy was performed 108 h after progesterone supplementation. In these patients only 14% (5/36) had an initially receptive result. Patients, in whom endometrial biopsy was performed 120 h after progesterone supplementation, showed a receptive endometrium in 42% (13/31) (*p* = 0.001).

Table 2 presents the categorized results of the endometrial receptivity analysis by the timing of biopsy.

**Table 2:** Test result of endometrial receptivity analysis categorized according to timing of biopsy

	biopsy after 108 h	biopsy after 120 h	*p*-value
N	36	31	
receptive	5 (14%)	13 (42%)	0.001^a^
non-receptive	31 (86%)	18 (58%)	0.001^a^
post-receptive	0 (0%)	1 (3%)	
pre-receptive	25 (69%)	11 (36%)	
early receptive	6 (17%)	6 (19%)	
second biopsy necessary	4 (11%)	0 (0%)	0.12^b^
P + 4.5	5 (14%)	1 (3%)	
P + 5	6 (17%)	13 (42%)	
P + 5.5	23 (64%)	6 (19%)	
P + 6	1 (3%)	11 (36%)	
P + 6.5	1 (3%)	0 (0%)	

*p*-value controlled with: ^a^ chi-squared test, ^b^ Fisher´s exact test

The comparison between personalized and standardized embryo transfer revealed no statistically significant different reproductive outcome (Table 1). CPR was 39% after personalized embryo transfer and 31% after standardized embryo transfer (*p* = 0.465), with an odds ratio of 1.4 (95% CI: 0.57, 3.41). Pregnancy rate and LBR were 49% and 27% after personalized and 44% and 19% after standardized embryo transfer, respectively. Biochemical pregnancy loss rate and clinical miscarriage rate were 21% and 31% after personalized and 29% and 40% after standardized embryo transfer, respectively. The proportion of patients who had a blastocyst transfer (personalized embryo transfer 79%, standardized embryo transfer 75%) and the proportion of patients having an embryo transfer with an embryo of good quality (personalized embryo transfer 73%, standardized embryo transfer 84%) was not significantly different.

In a further analysis, we divided the group of patients with a personalized embryo transfer into receptive and non-receptive patients. This comparison again showed no significant differences in the reproductive outcome after correction of progesterone timing (Table 3).

**Table 3:** Subgroup analysis of personalized embryo transfer (pET): Patient characteristics and clinical outcome—receptive vs non-receptive

	receptive *n* = 18	non-receptive *n* = 49	Odds Ratio (95% CI)^d^	*p*-value
Patient characteristics				
Age, years	36 (± 2)	35 (± 3)	—	0.42^b^
Body mass index, kg/m^2^	22 (± 5)	23 (± 4)	—	0.039^b^
AMH, ng/ml	3.3 (± 1.9)	3.0 (± 2.6)	—	0.26^b^
ICSI rate, *n* (%)	16 (89%)	43 (88%)	—	> 0.99^c^
Previous spontaneous clinical miscarriages, *n* (%)	4 (22%)	12 (25%)	—	> 0.99^c^
Previous ectopic pregnancies, *n* (%)	2 (11%)	5 (10%)	—	> 0.99^c^
Previous deliveries, *n* (%)	3 (17%)	3 (6%)	—	0.33^c^
Number of previous failed embryo transfers	4 (± 2)	5 (± 2)	—	0.20^b^
Time between scratching and transfer, days	121 (± 70)	115 (± 79)	—	0.53^b^
Clinical outcome				
Pregnancy rate, *n* (%)	6 (33%)	27 (55%)	0.4 (0.1–1.3)	0.11^a^
Clinical pregnancy rate, *n* (%)	5 (28%)	21 (43%)	0.5 (0.1–1.7)	0.26^a^
Live birth rate, *n* (%)	4 (22%)	14 (29%)	0.7 (0.2–2.6)	0.76^c^
Clinical miscarriage rate, *n* (%)	1/5 (20%)	7/21 (33%)	0.5 (0.1–5.4)	> 0.99^c^
Biochemical pregnancy loss rate, *n* (%)	1/6 (17%)	6/27 (22%)	0.7 (0.1–7.2)	> 0.99^c^
Embryo transfer using blastocyst, *n* (%)	15 (83%)	38 (78%)	—	0.74^c^
Good quality embryo, *n* (%)	11 (61%)	38 (78%)	—	0.22^c^

Numerical variables are reported as M (± SD) and categorical variables as n (%); *p*-value controlled with: ^a^ Chi-squared test, ^b^ Mann–Whitney U test, ^c^ Fisher´s exact test; ^d^ Odds Ratio for receptive vs. non-receptive, only computed for relevant clinical outcomes

Supplemental Table S1 presents the clinical outcome for each type of progesterone and shows no statistically significant difference.

There was no statistically significant difference when comparing the reproductive outcome between the group of patients who had the initial biopsy after 108 vs 120 h of progesterone administration (Supplemental Table S2).

## Discussion

A total of 73% of our study population had a displaced endometrial receptivity. The endometrial receptivity analysis was performed in our cohort after 108 to 120 h of progesterone supplementation. For the subgroup of patients who had the biopsy 120 h after progesterone initiation, the rate of displaced endometrial receptivity was still 58%.

When comparing the proportion of patients with a displaced endometrial receptivity between the studies published on this topic, one sees a wide spread between 18 to 85% [22, 24]. The reason for this large variation could be the observed patient population. It is suggested that patients who have been diagnosed with RIF and who have had a large number of unsuccessful embryo transfers are more likely to have a displaced endometrial receptivity than therapy-naïve patients. However, this is contradicted by the data of Patel et al., who demonstrated a displacement of endometrial receptivity in only 17.7% of patients in a RIF cohort with a history of four unsuccessful embryo transfers [24], which is even lower than the proportion of patients with displaced endometrial receptivity in a therapy-naïve patient population published by Simon and colleagues [17].

Although the performed test diagnosed a displaced endometrial receptivity in such a high percentage, a personalized embryo transfer did not increase the reproductive outcome compared to a standardized embryo transfer.

There could be different reasons for this:

First, it could be that the theory of a very narrow individual window of implantation in which progesterone exposure is assumed to be able to enhance or delay the endometrial maturity by precisely 12-h intervals is not true. In fact, a study in which day 3 embryos, warmed and cultured overnight to day 4 embryos, were transferred on the 3rd or 5th day of progesterone exposure in a programmed FET showed no significantly different clinical pregnancy rate [30]. This suggests that the window of implantation might be much larger than the manufacturers of the endometrial receptivity test assume.

Second, keeping in mind the data of Pirtea et al., namely that true implantation failure is present in less than 5% of patients after transfer of euploid embryos [1], it could be that the main cause of implantation failure in this cohort is not a displaced endometrial receptivity but aneuploidy of the embryos. Unfortunately, screening for aneuploidy (PGT-A) was not performed in our patient cohort, neither in the history nor in the study period, as this is prohibited by the German Embryo Protection Law. However, to counter the factor of aneuploidy, we set an age above 40 years as an exclusion criterion in the study design.

Nevertheless, the proportion of subjects with true non-embryo implantation failure in our cohort may be so small that personalized embryo transfer was the solution to the problem in only a few individual cases, but in most cases, there was an embryonic problem. However, if this theory is correct and displaced endometrial receptivity is not the main cause of recurrent implantation failure, the question arises as to why the proportion of patients with displaced endometrial receptivity is so high.

Third, in this context the doubts about the validity of the test discussed in reproductive community should be mentioned [18]. Many groups have found a different number of genes that were up or down regulated during the window of implantation [12, 14, 31]. It is debated that this alone indicates the inconsistency and dependence of the results on the methods used to study the genes, the complexity of the mathematical model, the methods and timing of the biopsy, the correction methods for the biopsy material, and the relationships to preovulatory progesterone levels [18].

Fourthly, it could be that the lack of statistical significance is due to the small population size. The a priori power analysis showed that for statistically significant evidence of an assumed increase in CPR of 10% by performing a personalized embryo transfer compared with a standardized embryo transfer, 356 subjects would have to be studied. Thus, to address the question of clinical relevance, analyses with significantly larger numbers of subjects are needed. Studies of such population size exclusively with RIF patients are rare, because true RIF (as already described above) is such a scarce diagnosis [1]. Our data may therefore also serve as a building block for further analyses to elucidate the benefits of a personalized embryo transfer guided by an endometrial receptivity analysis.

Comparable studies with RIF populations in which an endometrial receptivity analysis was performed and a personalized embryo transfer was compared with a standardized embryo transfer have been scarcely published and are conflicting. Most studies compared the clinical outcome of receptive versus non-receptive patients [21, 24, 25] without any control group and could also not find any difference between receptive and non-receptive patients. While Fodina et al. and Cozzolino et al. found no clinical benefit of an endometrial receptivity test for RIF patients in comparison to a control group [20, 32], a recently published study from the group of Jia et al. found, in contrast to our study, a significantly higher pregnancy and implantation rate in patients who underwent a personalized compared to a standardized embryo transfer [33]. However, it should be noted that ongoing pregnancy rate was not reported, and no clear distinction was made between programmed and natural FET cycles.

Our data further revealed a clinical miscarriage rate of 31–40%, which is considered very high and might be explained partly due to aneuploidy. However, as discussed previously, PGT-A is not allowed in Germany, so this could not be investigated. Among other reasons for miscarriage, the type of FET could also explain the high miscarriage rate. Although studies are conflicting, it is suspected that a programmed FET has a higher miscarriage rate than natural or modified natural FET cycles [34–36]. The cause of this could be the progesterone itself, as it is believed that in 25–33% of women who perform a programmed FET, higher serum progesterone levels are necessary for minimizing early pregnancy losses [37]. Individualization of the dosage and type of progesterone could therefore potentially have a positive effect on the miscarriage rate and consequently the LBR in a programmed cycle [38–40]. However, this was not performed in our cohort.

Limitations of the study are the retrospective design and the small-sized study population. Another limitation is the fact that the initial endometrial biopsy was not performed standardized after 120 h, but also after 108 h of progesterone supplementation. However, there was no statistically significant difference in reproductive outcome comparing patients who had the initial biopsy after 108 vs 120 h of progesterone supplementation.

Strengthens, on the other hand, is the fact, that only RIF patients in the case group, as well as in the control group, were examined, and not only the clinical pregnancy rate but even the more important parameter of LBR was reported.

## Conclusions

Patients with RIF have a high incidence of a displaced endometrial receptivity, according to the endometrial receptivity analysis. However, since reproductive outcome was not significantly higher in patients who performed a personalized embryo transfer, the displaced endometrial receptivity might not be the leading cause for the recurrent implantation failure in this population. The present study adds to the growing body of evidence, that a personalized embryo transfer guided by the result of an endometrial receptivity analysis might not be useful in patients with RIF; however, no general recommendations can be made based on these data alone.

## Supplementary information

Below is the link to the electronic supplementary material.

Supplementary file1 (DOCX 22 KB)

## Data Availability

The data supporting the findings of the study are available upon request.
