# Supplementary material for: One center experience with a personalized frozen-thawed embryo transfer in patients with recurrent implantation failure
Source: J Assist Reprod Genet. 2023 Jun 1;40(7):1639–47. doi: 10.1007/s10815-023-02835-7 (PMC10352213; doi:10.1007/s10815-023-02835-7)
Supplement: Supplementary file 1 — Supplementary file1 (DOCX 22 KB) [file 10815_2023_2835_MOESM1_ESM.docx]

**Supplemental Table S1**. Patient characteristics and clinical outcome after frozen-thawed embryo transfer (regardless whether it was executed personalized or standardized), divided by the type of progesterone.

|  | Crinone^®^  n=70 | Dydrogesterone  n=17 | MVP  n=9 | Prolutex^®^  n=3 | p-value |
| --- | --- | --- | --- | --- | --- |
| Patient characteristics |  |  |  |  |  |
| Age, years | 35.8 (± 2.8) | 33.6 (± 3.8) | 35.0 (± 2.3) | 37.7 (± 1.7) | 0.042^b^ |
| Body mass index, kg/m² | 23.2 (± 3.0) | 21.7 (± 2.7) | 21.4 (± 2.1) | 22.0 (± 3.3) | 0.44^b^ |
| AMH, ng/mL | 3.2 (± 2.4) | 3.0 (± 2.2) | 2.9 (± 2.4) | 2.9 (± 3.9) | 0.97^b^ |
| ICSI rate, n (%) | 66 (94.3%) | 14% (82.4%) | 7 (77.8%) | 1 (33.3%) | 0.004^a^ |
| Previous Spontaneous clinical miscarriages, n (%) | 14 (20%) | 2 (11.8%) | 2 (22.2%) | 1 (33.3%) | 0.78^a^ |
| Previous ectopic pregnancies, n (%) | 7 (10%) | 0 (0%) | 0 (0%) | 0 (0%) | 0.37^a^ |
| Previous deliveries, n (%) | 6 (8.6%) | 0 (0%) | 1 (11.1%) | 1 (33.3%) | 0.25^a^ |
| Number of previous failed embryo transfer | 4.1 (± 1.4) | 4.2 (± 2.0) | 4.7 (± 2.0) | 4.7 (± 1.2) | 0.76^b^ |
| Clinical outcome |  |  |  |  |  |
| Pregnancy rate, n (%) | 30 (42.9%) | 12 (70.6%) | 4 (44.4%) | 1 (33.3%) | 0.21^a^ |
| Clinical pregnancy rate, n (%) | 17 (24.3%) | 9 (52.9%) | 4 (44.4%) | 1 (33.3%) | 0.11^a^ |
| Live birth rate, n (%) | 15 (21.4%) | 5 (29.4%) | 4 (44.4%) | 0 (0%) | 0.32^a^ |
| Clinical miscarriage rate, n (%) | 7 (31.8%) | 4 (44.4%) | 0 (0%) | 1 (100%) | 0.21^a^ |
| Biochemical pregnancy loss rate, n (%) | 8 (26.7%) | 3 (25.0%) | 0 (0%) | 0 (0%) | 0.63^a^ |
| Embryo transfer using blastocyst, n (%) | 54 (77.1%) | 15 (81.2%) | 6 (66.7%) | 2 (66.7%) | 0.58^a^ |
| Good quality embryo rate, n (%) | 52 (74.3%) | 16 (94.1%) | 6 (66.7%) | 2 (66.7%) | 0.28^a^ |

Types of progesterone: Crinone^®^ 8% Gel vaginal, dydrogesterone 30 mg oral, micronized vaginal progesterone (MVP) 600 mg, Prolutex^®^ 25 mg subcutaneous.

Note: p-value controlled with: ^a^chi-squared test, ^b^one-factorial ANOVA with Bonferroni post-hoc test

**Supplemental Table S2.** Patient characteristics and clinical outcome categorized according to timing of initial biopsy after 108 versus 120 hours of progesterone supplementation.

|  | 108 hours  n=36 | 120 hours  n=31 | p-value |
| --- | --- | --- | --- |
| Patient characteristics | 36 | 31 |  |
| Age, years | 35 (±3) | 36 (±3) | 0.525^b^ |
| Body mass index, kg/m² | 24 (±4) | 23 (±5) | 0.336^b^ |
| AMH, ng/ml | 3.3 (±2,9) | 2.9 (±1.7) | 0.960^b^ |
| ICSI rate, n (%) | 34 (94%) | 25 (81%) | 0.131^c^ |
| Previous spontaneous clinical miscarriages, n (%) | 6 (17%) | 10 (32%) | 0.136^a^ |
| Previous ectopic pregnancies, n (%) | 4 (11%) | 3 (10%) | >0.999^c^ |
| Previous deliveries, n (%) | 1 (3%) | 5 (16%) | 0.088^c^ |
| Number of previous failed embryo transfers | 4.5 (±1.6) | 4.2 (±1.9) | 0.275^b^ |
| Time between scratching and transfer, days | 125 (±78) | 106 (±73) | 0.258^b^ |
| Clinical outcome |  |  |  |
| Pregnancy rate, n (%) | 18 (50%) | 15 (48%) | 0.895^a^ |
| Clinical pregnancy rate, n (%) | 11 (31%) | 11 (36%) | 0.668^a^ |
| Live birth rate, n (%) | 9 (25%) | 9 (29%) | 0.710^a^ |
| Clinical miscarriage rate, n (%) | 5/14 (36%) | 3/12 (25%) | 0.683^c^ |
| Biochemical pregnancy loss rate, n (%) | 4/18 (22%) | 3/15 (20%) | >0.999^c^ |
| Embryo transfer using blastocyst, n (%) | 25 (69%) | 28 (90%) | 0.036^a^ |
| Good quality embryo rate, n (%) | 26 (72%) | 23 (74%) | 0.856^a^ |

Note: p-value controlled with: ^a^chi-squared test, ^b^Mann-Whitney U test, ^c^Fisher´s exact test
